# Phylogeny and diversity of neotropical monkey lizards (Iguanidae: *Polychrus* Cuvier, 1817)

**DOI:** 10.1371/journal.pone.0178139

**Published:** 2017-06-01

**Authors:** Omar Torres-Carvajal, Claudia Koch, Pablo J. Venegas, Steve Poe

**Affiliations:** 1 Museo de Zoología, Escuela de Ciencias Biológicas, Pontificia Universidad Católica del Ecuador, Quito, Ecuador; 2 Zoologisches Forschungsmuseum Alexander Koenig, Bonn, Germany; 3 Centro de Ornitología y Biodiversidad (CORBIDI), Lima, Perú; 4 Department of Biology and Museum of Southwestern Biology, University of New Mexico, New Mexico, United States of America; University of Regina, CANADA

## Abstract

Neotropical monkey lizards (*Polychrus*) are arboreal lizards with compressed bodies, partially fused eyelids and strikingly long, whip-like tails. The eight currently recognized species occur in the lowlands of South and Central America. Based on the largest taxon and character sampling to date, we analyze three mitochondrial and one nuclear gene using Bayesian methods to (1) infer the phylogeny of *Polychrus* under both concatenated-tree and species-tree methods; (2) identify lineages that could represent putative undescribed species; and (3) estimate divergence times. Our species tree places *P*. *acutirostris* as the sister taxon to all other species of *Polychrus*. While the phylogenetic position of *P*. *gutturosus* and *P*. *peruvianus* is poorly resolved, *P*. *marmoratus* and *P*. *femoralis* are strongly supported as sister to *P*. *liogaster* and *P*. *jacquelinae*, respectively. Recognition of *P*. *auduboni* and *P*. *marmoratus* sensu stricto as distinct species indicates that the populations of "*P*. *marmoratus*" from the Amazon and the Atlantic coast in Brazil represent separate species. Similarly, populations of *P*. *femoralis* from the Tumbes region might belong to a cryptic undescribed species. Relative divergence times and published age estimates suggest that the orogeny of the Andes did not play a significant role in the early evolution of *Polychrus*.

## Introduction

Neotropical monkey lizards *Polychrus* Cuvier, 1817 are restricted to South America on both sides of the Andes, except for *P*. *gutturosus* Berthold, 1845, which ranges from the Pacific coast in Ecuador and Colombia into Central America as far north as Nicaragua. Two species, *P*. *gutturosus* and *P*. *auduboni* Murphy et al., 2017, have colonized islands off the coast of South America—Gorgona Island in Colombia and Trinidad and Tobago, respectively [1, 2]. The eight recognized species [1, 3] of monkey lizards are remarkable among New World lizards in that they resemble Old World chameleons in both morphology and behavior [3–5]; they are arboreal, slow-moving lizards with a laterally compressed body and cone-shaped eyes with partially fused eyelids [6]. Unlike chameleons, however, monkey lizards have long limbs and digits, as well as strikingly long whip-like tails.

The phylogenetic position of *Polychrus* relative to other clades of iguanid lizards also ranked traditionally as genera is currently disputed. The first major phylogenetic analysis of iguanid lizards based on morphology placed *Polychrus* within a clade named "*the anoloids*" as sister to all other species in that clade [7]. Later, the "*anoloids"* were ranked as a family, the Polychridae (= Polychrotidae), where *Polychrus* was recovered as sister to *Anolis* [8]. Twelve years later, the monophyly of Polychrotidae was rejected by a combined (i.e., morphology and DNA sequence data) phylogenetic analysis, and the name Polychrotidae was restricted to the clade containing *Polychrus* and *Anolis* [9], although that clade was not supported by the molecular evidence alone. By contrast, although the original clade Polychrotidae (*the anoloids*) was again found not to be monophyletic by a subsequent combined analysis, its monophyly could not be statistically rejected [10]. In that study, *Polychrus* was estimated to be sister to (1) *Anolis* (morphological evidence), (2) all other iguanids or tropidurines (DNA evidence), or (3) hoplocercines (combined evidence) depending on which dataset was analyzed. Subsequent phylogenetic analyses of iguanids based on a larger number of morphological characters and inclusion of fossil taxa [11, 12] supported monophyly of Polychrotidae (= Polychrotinae of [10, 13]). By contrast, phylogenetic studies based solely on DNA sequence data of < 10 loci [14, 15] have failed to recover the monophyly of Polychrotidae, both sensu Frost and Etheridge (1989) and sensu Frost et al. (2001; i.e., *Polychrus* + *Anolis*). More recently, Townsend et al. [16] analyzed the phylogenetic relationships among iguanids using 29 nuclear loci. Although these authors failed to statistically reject the monophyly of Polychrotidae sensu Frost et al. (2001), they also found strong evidence for the polyphyly of this clade and thus proposed to restrict the name Polychrotidae to *Polychrus*, whereas the name Dactyloidae was resurrected to include *Anolis* (sensu [17]). Subsequent large-scale phylogenetic analyses have yielded conflicting results. The squamate molecular phylogeny presented by Pyron et al. [18] for 4000+ species shows *Polychrus* as sister to hoplocercines and *Anolis* as sister to corytophanines, whereas molecular phylogenies including more loci but a reduced number of squamate taxa [19, 20] support a sister taxon relationship between *Polychrus* and *Anolis* (i.e., Polychrotidae sensu Frost et al. 2001). The *Polychrus-Anolis* sister relationship is strongly supported in a recent phylogeny based on 691 morphological characters and 46 genes for 161 living and 49 fossil squamate taxa [21]. As noted by other authors [16, 18], whether they are sister taxa or not, the monophyly of both *Polychrus* and *Anolis* (sensu [17, 22]) is strongly supported by all published studies.

The most comprehensive phylogeny of monkey lizards was presented by Frost et al. (2001) as part of a phylogenetic analysis of the "Polychrotidae" sensu Frost and Etheridge (1989). Using morphological data of one specimen each of all six species of *Polychrus* recognized at that time (i.e., excluding *P*. *jacquelinae* Koch et al., 2011 and *P*. *auduboni*), as well as 12S-tRNA^Val^ -16S sequences of four species (*P*. *liogaster* Boulenger, 1908 and *P*. *peruvianus* Noble, 1924 excluded), Frost et al. (2001) recovered a monophyletic *Polychrus*. Nonetheless, the different datasets (morphology, DNA and combined) yielded conflicting relationships among species. Surprisingly, with the notable exception of the complete mitochondrial genome of *P*. *marmoratus* Linnaeus, 1758 [23], the only additional sequences generated in mtDNA-based phylogenetic studies including species of *Polychrus* are two ND2 gene fragments of two species [10, 24] and, more recently, 16 COI and 16S sequences of *P*. *marmoratus* [1]. Nuclear DNA sequences of 40 and 5 loci were generated for *P*. *marmoratus* and *P*. *liogaster*, respectively, in different phylogenetic studies not focused on the phylogeny of *Polychrus* [16, 20, 25, 26]. Based on data available in Genbank, Pyron et al. [18] obtained a different *Polychrus* tree topology from the hypotheses presented by Frost et al. (2001) about a decade earlier; however, this discrepancy might be a consequence of misidentifying one ND2 sample of *P*. *gutturosus* as *P*. *acutirostris* (see Table 1).

**Table 1:** Vouchers, locality data, and GenBank accession numbers of taxa and gene regions included in this study.

Taxon	Voucher and locality	Lat	Long	GenBank accession number			
				12S	16S	ND2	RAG1
***Polychrus***							
*acutirostris*	**MVZ 230130**; Pet trade specimen with no locality data.	–	–	–	KY458424	–	–
*acutirostris*	**POE 2767**; Bolivia: Warnes: in fields around Hotel Rio Sehra Resort.	-17.56	-63.19	KY982473*	KY982367*	KY982409*	KY982442*
*acutirostris*	**POE 2772**; Bolivia: Warnes: in fields around Hotel Rio Sehra Resort.	-17.56	-63.19	KY982474*	KY982368*	–	KY982443*
*acutirostris*	**POE 2783**; Bolivia: Ichilo: Trails around Ambero EcoResort.	-17.45	-63.67	KY982475*	KY982369*	–	KY982444*
*acutirostris*	**UNNEC 1368**; Argentina: Formosa: Pilcomayo: Cnia. Primavera.	-25.21	-58.29	AF338331^b^	–	–	–
*acutirostris*	**ZFMK 38742**; Bolivia: Tarija: Chaco: Villa Montes.	-21.25	-63.45	KY982476*	KY982370*	KY982410*	–
*acutirostris*	**ZFMK 59756**; Brazil: Pernambuco: Close to Caruaru.	-8.28	-35.97	KY982477*	KY982371*	KY982411*	–
*auduboni*	**CAS 231770**; Trinidad and Tobago: Trinidad, Nariva Road, Manzanilla Beach.	10.49	-61.05	–	KY458419	–	–
*auduboni*	**CAS 231781**; Trinidad and Tobago: Trinidad, 5 km E of Laguna Mar Beach Resort, Blanchisseuse.	10.79	-61.30	–	KY458420	–	–
*auduboni*	**LSUMZ 4458**; Trinidad and Tobago: Trinidad, San Fernando.	10.28	-61.45	–	KY458416	–	–
*auduboni*	**RML unnumbered**; Trinidad and Tobago: Tobago, west side of Charlotteville.	–	–	–	KY458422	–	–
*auduboni*	**UWIZM.2012.42.12**; Trinidad and Tobago: Tobago.	–	–	–	KY458411	–	–
*auduboni*	**UWIZM.2012.27.47**; Trinidad and Tobago: Tobago, Arnos Valle Bridge Courtland River.	11.21	-60.76	–	KY458417	–	–
*auduboni*	**UWIZM.2012.27.61**; Trinidad and Tobago: Trinidad, Arima Valley.	10.68	-61.28	–	KY458418	–	–
*auduboni*	**ZFMK 74419**; Venezuela: Bolívar: Guri Barrier Lake, Guri.	7.52	-62.97	KY982478*	KY982372	KY982412*	–*
*femoralis*	**CORBIDI 4220**; Peru: Piura: Huancabamba: Chigña Alta (Huarmaca).	-5.58	-79.67	KY982479*	KY982373	KY982413*	KY982445*
*femoralis*	**CORBIDI 4221**; Peru: Piura: Huancabamba: Chigña Alta (Huarmaca).	-5.58	-79.67	KY982480*	KY982374	KY982414*	KY982446*
*femoralis*	**CORBIDI 7944**; Peru: Tumbes: Tumbes: Quebrada Huarapal-Angostura.	-3.78	-80.34	KY982481	KY982375	KY982415*	–
*femoralis*	**CORBIDI 7947**; Peru: Tumbes: Quebrada Faical-El Caucho.	-3.82	-80.27	KY982482	KY982376	KY982416*	KY982447*
*femoralis*	**KU 218381**; Ecuador: Manabí: 1.5 km S Puerto Cayo.	-1.36	-80.74	AF338335^b^	–	–	–
*femoralis*	**QCAZ 10521**; Ecuador: Santa Elena: Ecuasal pools.	-2.02	-80.70	KY982486*	KY982380*	KY982420*	KY982451*
*femoralis*	**QCAZ 10583**; Ecuador: Manabí: El Aromo.	-1.05	-80.83	KY982487*	KY982381*	KY982421*	KY982452*
*femoralis*	**QCAZ 11477**; Ecuador: Manabí: Bahía de Caráquez, Reserva Biológica Cerro Seco.	-0.61	-80.44	KY982488*	KY982382*	KY982422*	KY982453*
*femoralis*	**QCAZ 4478**; Ecuador: Loja: Bella María community on road Cariamanga-Gonzanamá.	-4.18	-79.60	KY982483*	KY982377*	KY982417*	KY982448*
*femoralis*	**QCAZ 6714**; Ecuador: Loja: Puyango Protected Forest.	-3.88	-80.08	KY982484*	KY982378*	KY982418*	KY982449*
*femoralis*	**QCAZ 9150**; Ecuador: Guayas: Cerro Blanco Protected Forest.	-2.18	-80.02	KY982485*	KY982379*	KY982419*	KY982450*
*femoralis*	**ZFMK 85032**; Peru: Lambayeque: Chaparri.	-6.51	-79.46	KY982489*	KY982383*	KY982423*	KY982454*
*gutturosus*	**AMNH 10182**; voucher number in error; unknown locality^f^.	–	–	–	–	AF055925^a^	–
*gutturosus*	**OMNH**; No further data available.	–	–	AF338338^b^	–	–	–
*gutturosus*	**POE 1633**; Panama: Veraguas: Santa Fe: Altas Piedras, about 10 km NW Santa Fe.	8.61	-81.19	KY982490*	KY982384*	–	–
*gutturosus*	**POE 1884**; Panama: Coclé: 30 km NE of Penonome, Posada Ecología Hotel.	8.68	-80.21	KY982491*	KY982385*	–	–
*gutturosus*	**MCZ R-186149**; Costa Rica: San José: Piedras Negras, Río Virilla.	9.92	-84.32	KY982492*	KY982386*	–	–
*gutturosus*	**QCAZ 5710**; Ecuador: Pichincha: La Unión del Toachi.	-0.32	-78.96	KY982493*	KY982387*	KY982424*	KY982455*
*gutturosus*	**QCAZ 8940**; Ecuador: Esmeraldas: road Caimito-Quingue.	0.72	-80.09	KY982494*	KY982388*	KY982425*	KY982456*
*gutturosus*	**QCAZ 9788**; Ecuador: Santo Domingo de los Tsáchilas: 6.9 km from Santo Domingo.	-0.33	-79.22	KY982495*	KY982389*	KY982426*	KY982457*
*gutturosus*	**ZFMK 25729**; Pet trade specimen from Central America.	–	–	KY982496*	KY982390*	KY982427*	KY982458*
*gutturosus*	**ZFMK 40832**; Costa Rica: Puntarenas: Palmar.	8.96	-83.50	KY982497*	KY982391*	–	–
*jacquelinae*	**CORBIDI 7724**; Peru: La Libertad: Bolívar: San Vicente/Pusac.	-6.98	-77.90	KY982498*	KY982392*	KY982428*	KY982459*
*jacquelinae*	**CORBIDI 7725**; Peru: La Libertad: Bolívar: San Vicente/Pusac.	-6.98	-77.90	KY982499*	KY982393*	KY982429*	–
*jacquelinae*	**ZFMK 91764**; Peru: La Libertad: Bolívar: San Vicente/Pusac.	-6.98	-77.90	KY982500*	KY982394*	KY982430*	KY982460*
*liogaster*	**CORBIDI 9782**; Peru: Cusco: La Convención: KP55, Bajo Puyantimarí.	-12.21	-73.01	KY982501*	KY982395*	KY982431*	KY982461*
*liogaster*	**POE 2758**; Bolivia: Marbán: between Loreto & Camiaco.	-15.33	-64.86	KY982502*	KY982396*	–	–
*liogaster*	**POE 2782**; Bolivia: Ichilo: Trails around Ambero EcoResort.	-17.45	-63.67	KY982503*	KY982397*	–	KY982462*
*liogaster*	**ZFMK 80027**; Bolivia: Santa Cruz: 13 km W Yapacaní.	-17.40	-64.00	KY982504*	KY982398*	KY982432*	KY982463*
*marmoratus*	**AMNH 138080**; Guyana: Northern Rupununi Savanna, Yupukari (on Rupununi River), 7 mi (airline) SSW Karanambo.	–	–	–	KY458410	–	–
*marmoratus*	**AMNH 139787**; Guyana: Southern Rupununi Savanna, Aishalton (on Kubanawau Creek).	2.48	-59.32	AF338329^b^	–	–	–
aff. *marmoratus*	Pet trade specimen with no data.	–	–	NC_012839^d^	NC_012839^d^	NC_012839^d^	–
aff. *marmoratus*	**KU 212631**; Peru: San Martín: 14 km ESE of Shapaja.	-6.62	-76.18	–	KY458413	–	–
aff. *marmoratus*	**LSUMZ 14270;** Brazil: Para: Agropecuaria Treviso, LTDA, ca 101 km south, 18 km east Santarem.	-3.15	-54.84	–	KY458415	–	–
aff. *marmoratus*	**LSUMZ 14271;** Brazil: Para: Agropecuaria Treviso, LTDA, ca 101 km south, 18 km east Santarem.	-3.15	-54.84	–	KY458412	–	–
aff. *marmoratus*	**LSUMZ 14392;** Brazil: Para: Agropecuaria Treviso, LTDA, ca 101 km south, 18 km east Santarem.	-3.15	-54.84	–	KY458414	–	–
aff. *marmoratus*	**MVZ 163071**; Peru, Amazonas, vicinity of Sua (Aguaruna village), Río Cenepa (4°34'12.00''S, 78°13'18.01''W)	-4.57	-78.22	–	KY458423	–	–
aff. *marmoratus*	**OU 36693**; Brazil: Pará: ~101 km S and 18 km E Santarem, Agropecuaria Treviso LTDA.	-3.15	-54.84	–	–	AF528738^e^	–
aff. *marmoratus*	**QCAZ 10149**; Ecuador: Morona Santiago: road Macas-Limón.	-2.76	-78.31	KY982505*	KY982399*	KY982433*	KY982464*
aff. *marmoratus*	**QCAZ 10223**; Ecuador: Sucumbíos: 1.6 km S Jivino Verde.	-0.19	-76.83	KY982506*	KY982400*	KY982434*	KY982465*
*peruvianus*	**CORBIDI 5724**; Peru: Cajamarca: Jaén: Pucará.	-6.04	-79.13	–	KY982401*	KY982435*	KY982466*
*peruvianus*	**CORBIDI 5727**; Peru: Cajamarca: Jaén: Bellavista.	-5.64	-78.66	KY982507*	KY982402*	KY982436*	KY982467*
*peruvianus*	**CORBIDI 5736**; Peru: Amazonas: Utcubamba: Puerto Malleta.	-6.06	-78.60	KY982508*	KY982403*	KY982437*	KY982468*
*peruvianus*	**CORBIDI 5739**; Peru: Cajamarca: Cutervo: Lucuma.	-6.07	-78.61	–	KY982404*	KY982438*	KY982469*
*peruvianus*	**ZFMK 88709**; Peru: Cajamarca: Perico.	-5.35	-78.79	KY982509*	KY982405*	KY982439*	KY982470*
*peruvianus*	**ZFMK 88713**; Peru: Amazonas: Bagua Grande.	-5.79	-78.38	KY982510*	KY982406*	KY982440*	KY982471*
*peruvianus*	**ZFMK 90832**; Peru: Amazonas: Cumba.	-5.94	-78.65	KY982511*	KY982407*	KY982441*	–
**Outgroups**							
*Anolis carolinensis*	**–**	–	–	NC_010972	NC_010972	NC_010972	FJ356739
*Basiliscus plumifrons*	**–**	–	–	–	–	–	AY662599
*Basiliscus vittatus*	**–**	**–**	**–**	AB218883	AB218883	AB218883	–
*Brookesia decaryi*	**–**	**–**	**–**	AB474914	AB474914	AB474914	FJ984238
*Enyalioides laticeps*	**–**	–	–	KY982512*	KY982408*	EU586748	EU586773
*Gambelia wislizenii*	**–**	**–**	**–**	NC_012831	NC_012831	NC_012831	AY662600
*Iguana iguana*	**–**	**–**	**–**	AJ278511	AJ278511	AJ278511	–
*Leiocephalus personatus*	**–**	**–**	**–**	NC_012834	NC_012834	NC_012834	–
*Leiocephalus raviceps*	**–**	**–**	**–**	–	–	–	FJ356744
*Liolaemus lineomaculatus*	**–**	**–**	**–**	–	–	–	FJ356740
*Liolaemus scapularis*	**–**	**–**	**–**	DQ237595	L41447	AF099258	–
*Oplurus cuvieri*	**–**	**–**	**–**	U39587	AF215260	U82685	AY662601
*Phrynosoma cornutum*	**–**	**–**	**–**	DQ385390	L41453	DQ385344	FJ356738
*Phymaturus somuncurensis*	**–**	**–**	**–**	JX969089	AF215261	AF049865	AY662594
*Pristidactylus scapulatus*	**–**	**–**	**–**	AF338333	–	AF528732	FJ356746
*Pristidactylus torquatus*	**–**	**–**	**–**	–	L41456	–	–
*Stenocercus guentheri*	**–**	**–**	**–**	–	L41481	DQ080223	KY982472*
*Stenocercus roseiventris*	**–**	**–**	**–**	AF362522	–	–	–
*Uromastyx benti*	**–**	–	–	AB114447	AB114447	AB114447	FJ356733

Latitude (Lat) and Longitude (Long) data are in decimal degrees (WGS84). Voucher and locality data of outgroup taxa are not provided. Asterisks indicate new sequences obtained for this study. AMNH (American Museum of Natural History–Herpetology Collection, USA), CAS (California Academy of Sciences, USA), CORBIDI (División de Herpetología, Centro de Ornitología y Biodiversidad, Lima, Peru), KU (University of Kansas Biodiversity Institute-Herpetology Collection, Kansas, USA), LSUMZ (Lousiana State University Museum of Natural Science, USA), MCZ R (Museum of Comparative Zoology-Reptile collection, University of Harvard, USA), MVZ (Museum of Vertebrate Zoology, University of California, Berkeley, USA), POE (Steve Poe field number, University of New Mexico, USA), QCAZ (Museo de Zoología, Pontificia Universidad Católica del Ecuador, Quito, Ecuador), UNNEC (Universidad Nacional del Nordeste, Argentina), UWIZM (University of the West Indies Zoology Museum), ZFMK (Zoologisches Forschungsmuseum Alexander Koenig, Germany).

^a^Jackman et al. (1999)

^b^Frost et al. (2001)

^c^Hass et al. (1993)

^d^Mitochondrial complete genome (Okajima and Kumazawa 2009)

^e^Schulte et al. (2003)

^f^This sample was originally misidentified as *P*. *acutirostris*.

**Fig 1:**
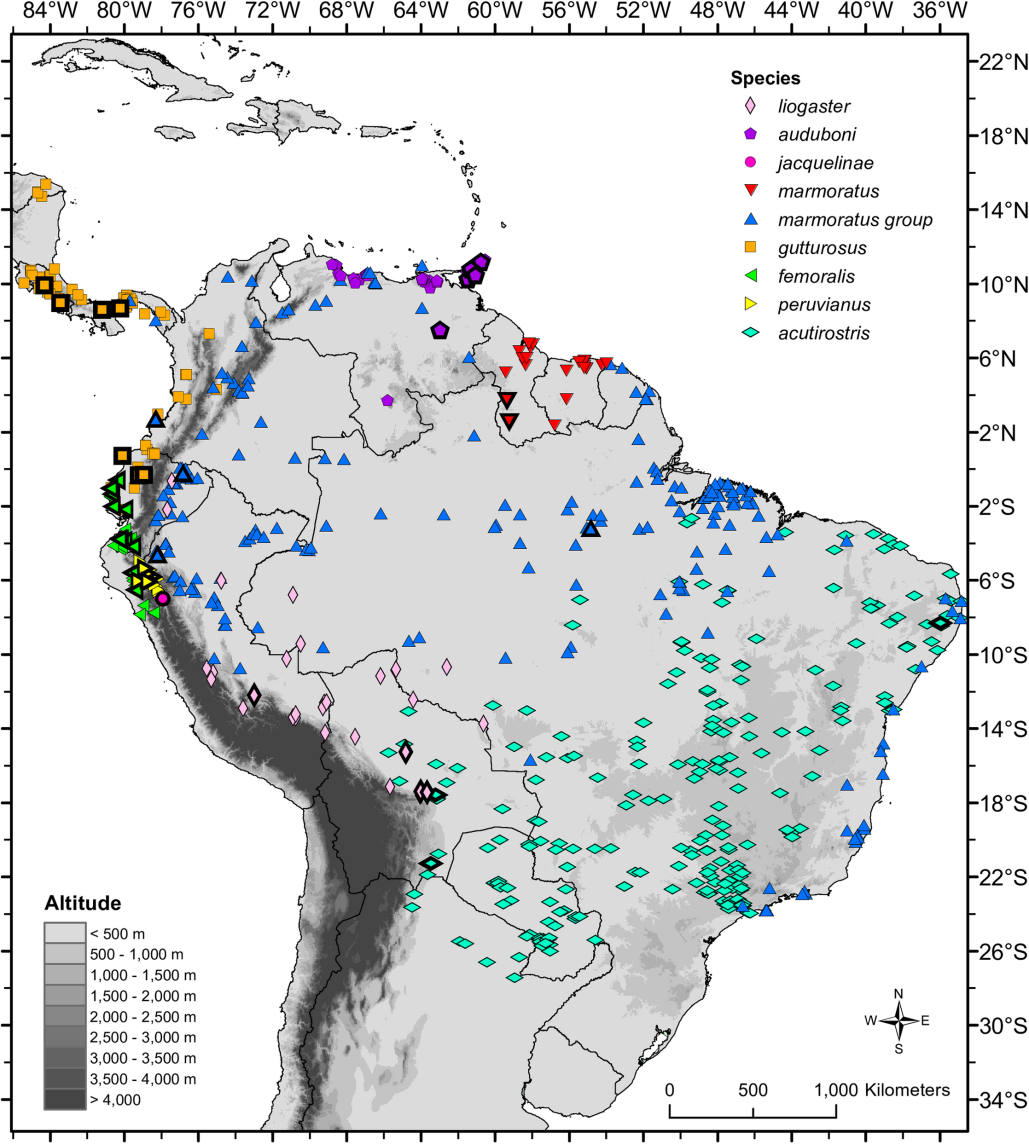
Distribution of *Polychrus* lizards. Bold-black delimited symbols represent localities from which DNA was used in our analyses. In accordance with Murphy et al. (2017) we restrict the name *P*. *marmoratus* to the populations in Guyana and Suriname and designate remaining populations as the “*marmoratus*” group. Except for *P*. *femoralis*, most locality data derived from the literature and unverified VertNet records.

Thus, in spite of its relatively low diversity (8 species), a molecular phylogeny of *Polychrus* based on a complete dataset of more than two mitochondrial genes and more than four species has not been published. Moreover, no attempts have been made to explore the genetic variation and diversity within *Polychrus* despite the wide distribution of most species (Fig 1). In this paper we analyze the phylogenetic relationships among all currently recognized species of monkey lizards based on broad geographic sampling. Using one nuclear and three mitochondrial genes, we (1) test the monophyly of *Polychrus* and its currently recognized species based on the largest taxon and character sampling to date; (2) identify lineages that could represent putative undescribed species; and (3) co-estimate divergence times and a species tree of *Polychrus* under a coalescent model.

## Materials and methods

### Fieldwork and data sampling

A total of 35 specimens representing different species of *Polychrus* were collected during several field trips to different localities in Bolivia, Ecuador, Costa Rica, Panama, and Peru. After lethal anesthetization of voucher specimens with an intracoelomic injection of Nembutal or T61®, tissue samples were taken from the thigh muscle and the specimens were stored in 70% ethanol and deposited in the collections of the Museo de Zoología de la Pontificia Universidad Católica (QCAZ), Quito, Ecuador; the Centro de Ornitología y Biodiversidad (CORBIDI), Lima, Peru; the Zoologisches Forschungsmuseum Alexander Koenig (ZFMK), Bonn, Germany; the Museum of Southwestern Biology (MSB; POE field numbers) at the University of New Mexico, Albuquerque, United States; and the Museum of Comparative Zoology at Harvard University, Cambridge, United States. Six additional tissue samples were taken from specimens previously housed at ZFMK (Table 1).

We used ArcGis to generate a distribution map of all species of *Polychrus* based on locality data from databases of the collections listed above, as well as data from the literature and VertNet (www.vertnet.org).

Voucher specimens and tissue samples were obtained following ethical and technical protocols [27]. Collecting and export permits were kindly provided by the Ministerio de Agricultura of Peru (collecting: 071–2007–INRENA–IFFS–DCB, 0020–2009–AG–DGFFS–DGEFFS, 0424–2010–AG–DGFFS–DGEFFS; export: 0017799–AG–INRENA, 001829–AG–DGFFS, 003983–AG–DGFFS), Ministerio de Ambiente of Ecuador (001–10 IC-FAU-DNB/MA, 005-12-IC-FAU-DNB/MA, 008–09 IC-FAU-DNB/MA), Autoridad Nacional del Ambiente (ANAM) of Panama (A-135-03), and Ministerio del Ambiente y Energia (MINAE) of Costa Rica (217-2008-SINAC). Approval by an Ethics Committee for collecting lizard specimens and tissue samples is not required by CORBIDI, QCAZ, and ZFMK. However, this study was evaluated and approved by the DGA (Dirección General Académica) of the Pontificia Universidad Católica del Ecuador in accordance with the guidelines for environmental and social impacts of research projects. The DGA committee evaluates projects to determine observance of its norms for ethical scientific research. Genetic data for Ecuadorian specimens were obtained under the Genetic Resources Access Contract No MAE-DNB-CM-2015-0025 issued by Ministerio de Ambiente del Ecuador to Pontificia Universidad Católica del Ecuador. Research at MSB was carried out under protocol number 16-200554-MC, approved by the Institutional Animal Care and Use Committee at the University of New Mexico.

### Laboratory protocols

We obtained nucleotide (nt) sequences from three mitochondrial genes, ribosomal small (12S, 427 nt) and large (16S, 563 nt) subunit genes, subunit II of NADH dehydrogenase (ND2, 1038 nt), as well as one nuclear gene, recombination activating gene (RAG1, 1027 nt). For these genes we generated novel DNA sequences from 41 specimens representing all currently recognized species of *Polychrus* (Fig 1, Table 1), as well as one specimen each of *Enyalioides laticeps* (12S and 16S) and *Stenocercus guentheri* (RAG1). In addition, we obtained sequences from GenBank representing 14 major clades of iguanian lizards (Table 1; [7, 8, 10, 13]).

Genomic DNA was isolated from frozen muscle or liver tissues using a guanidinium isothiocyanate extraction protocol. Polymerase Chain Reaction (PCR) amplification of gene fragments was performed in a final volume of 25 μl reactions using 1X PCR Buffer (–Mg), 3 mM MgCl_2_, 0.2 mM dNTP mix, 0.2 μM of each primer, 0.1 U/μl of Platinum® *Taq* DNA Polymerase (Invitrogen, Carlsbad, CA) and 1 μl of extracted DNA. Negative controls were run on all amplifications to check for contamination. Primers and PCR amplification protocols are presented in Table 2. Polymerase chain reaction products were analyzed on 1% agarose gels by horizontal electrophoresis (the target fragment size was estimated from molecular weight markers), using SYBR® Safe (Invitrogen, Carlsbad, CA) staining, and analyzed with a Molecular Imager® Gel Doc^TM^ XR+ Imaging System (Bio Rad, Hercules, CA). Amplified products were treated with ExoSAP-IT (Affymetrix, Cleveland, OH) to remove remaining dNTPs and primers, and extraneous single-stranded DNA produced in the PCR. Double stranded sequencing of the PCR products were performed in both directions by Macrogen Inc. New sequences were deposited in GenBank (Table 1).

**Table 2:** Primers and protocols used for amplification and sequencing reactions.

Gene	Primers 5'–3' sequence	Source	PCR protocol
12S	• F: CTGGGATTAGATACCCCACTA • F: AAACTGGGATTAGATACCCCACTAT • R: TGAGGAGGGTGACGGGCGGT • R: TGAGGAGGGTGACGGGCGGT	Harris et al. [28]; Kocher et al. [29]	• 96°C (3:00), 40 x (95°C (0:30), 52°C (1:00), 72°C (1:00)), 72°C (10:00) • 94°C (1:30), 38 x (94°C (0:45), 50°C (1:00), 74°C (2:00)), 74°C (5:00)
16S	• F : CGCCTGTTTATCAAAAACAT • R : GAGGGTGACGGGCGGTGTGT	Palumbi et al. [30]	95°C (15:00), 15 x (94°C (0:35), 60°C (1:30), 72°C (1:30)) + 25 x (94°C (0:35), 45°C (1:30), 72°C (1:30)), 72°C (10:00)
ND2	• F: CGATTCCGATATGACCARCT • F: CATACCCWCGATTYCGATAYGATC • F: AAGCTWTCGGGCCCATACC • R: TTGGGTAKTTAGCTGTTAA • R: GGGCCCATACCCCNAANATG	Kumazawa and Nishida [31]; Macey et al. [32]; this study	• 94°C (2:00), 25 x (94°C (0:30), 52°C (0:30), 72°C (2:30)), 72°C (10:00) • 95°C (15:00), 15 x (94°C (0:35), 60°C (1:30), 72°C (1:30)) + 25 x (94°C (0:35), 45°C (1:30), 72°C (1:30)), 72°C (10:00)
RAG 1	• F: CAAAGTRAGATCACTTGAGAAGC • R: ACTTGYAGCTTGAGTTCTCTTAGRCG• R: AGCTTGAGTTCTCTTAGRC	Schulte and Cartwright (2009)	• 94°C (2:00), 25 x (94°C (0:30), 52°C (0:30), 72°C (2:30)), 72°C (10:00) • 95°C (15:00), 40 x (94°C (0:20), 60°C (0:50), 72°C (1:30)), 72°C (10:00)

F = forward; R = reverse.

### Alignment, model selection, and phylogenetic analyses

Data were assembled and aligned in Geneious v9 [33] under default settings for the alignment program MAFFT [34]. Ribosomal (12S and 16S) gene regions with multiple gaps were realigned to minimize indels and optimize nucleotide identities among different individuals. ND2 and RAG1 sequences were translated into amino acids for confirmation of alignment. The best-fit nucleotide substitution models and partitioning scheme were chosen simultaneously using PartitionFinder v1.1.1 [35] under the Bayesian Information Criterion (BIC). The “greedy” algorithm was used with branch lengths of alternative partitions “linked” to search for the best-fit scheme.

A Bayesian inference method was used to obtain the optimal tree topology of the combined, partitioned dataset using MrBayes v3.2.1 [36]. All parameters except topology and branch lengths were unlinked between partitions, and rate variation (prset ratepr = variable) was invoked. Four independent runs, each with four MCMC chains, were run for 10^7^ generations, sampling every 1,000 generations. Results were analyzed in Tracer v1.6 [37] to assess convergence and effective sample sizes (ESS) for all parameters. Additionally, we verified that the average standard deviation of split frequencies between chains and the potential scale reduction factor (PSRF) of all the estimated parameters approached values of ≤ 0.01 and 1, respectively. Of the 10,000 trees resulting per run, 25% were discarded as “burn-in”. The resultant 30,000 trees were used to calculate posterior probabilities (PP) for each bipartition in a maximum clade credibility tree in TreeAnnotator v1.8.3 [38]. Phylogenetic trees were rooted with the acrodont iguanians *Brookesia* and *Uromastyx* [39], visualized and edited using FigTree v1.4.2 [40].

### Chronophylogenetic analysis

We estimated a *Polychrus* species tree from the mitochondrial and nuclear trees under a coalescent model–and simultaneously estimated relative divergence times–using the Starbeast method (Heled & Drummond, 2010) implemented in Beast 1.8.3. For this analysis we included only species of *Polychrus* (i.e., tree root was estimated by the clock model [41]). Models of nucleotide substitution and partition scheme were selected in PartitionFinder as explained above. The analyses were conducted under a model with uncorrelated substitution rates among branches and the rate for each branch independently drawn from an underlying lognormal distribution (Drummond et al., 2006). Because our sampling of the "*marmoratus*" species complex (i.e., including *P*. *auduboni*) was limited (Fig 1), we considered this complex as a single species for this analysis.

Previous studies differing in gene data, taxon sampling and analytical methods have produced a wide range of age estimates and sister taxa for *Polychrus* (Table 3). Therefore, here we consider that reliable internal and nearby external calibrations are not yet available. To reflect the absence of calibration dates, default parameter priors were used except for the *mean of branch rates* parameter (ucld.mean), which was fixed to 1.0 resulting in time being measured in units that have been arbitrarily chosen so that 1 time unit corresponds to the mean time required for the accumulation of 1 substitution per site (Drummond et al., 2006; Drummond and Rambaut, 2007). Search parameters and tree construction were similar to the Bayesian analysis described above, with three runs and a 'Yule Process' species tree prior under the 'Piecewise linear & constant root' population size model. Results were analyzed in Tracer v1.6 [37] to assess convergence and effective sample sizes (ESS) for all parameters. All phylogenetic analyses were carried out in the CIPRES Science Gateway [42].

**Table 3:** Estimated ages of *Polychrus* from the literature.

N	age	sister taxon	BPP	BB	Br	Reference
3	~32	*Anolis*	0.31	—	—	Pyron [43]
1	~45	(*Corytophanes*, *Basiliscus*)	0.95	—	—	Pyron [43]
3	~55	(Leiosaurinae, Anisolepinae, *Afairiguana*)	—	—	1	Conrad et al. [11]
2	~61	*Chalarodon*, (*Leiosaurus*, *Urostrophus*)	0.38	—	—	Prates et al. [26]
1	~62	*Leiocephalus*	0.55/0.88	15	—	Townsend et al. [16]
4	78.11	Dactyloidae	—	58	—	Zheng and Wiens [44]
1	~125	*Leiocephalus*	<0.95	<70	—	Noonan & Sites [14]

For each reference, number of species of *Polychrus* included in the analysis (N), estimated age of *Polychrus* in Myr, sister taxon to *Polychrus*, and branch support value for the sister taxon relationship (BPP = Bayesian posterior probabilities, BB = Bootstrap support values, Br = Bremer support values) are given. Approximate ages are based on time scale bars when the exact date was not specified in the reference.

### Species delimitation analysis

We identified clades or single branches within currently recognized species of *Polychrus* as putative species if (1) branches were much longer with respect to other branches within the clade corresponding to the currently recognized species (see below), and (2) their geographic distribution was disjunct with respect to other terminals within the currently recognized species.

We evaluated diagnosability and monophyly of putative species using the Species Delimitation plugin [45] in Geneious 7.1.9 [33]. We calculated (1) the mean probability of correctly identifying an unknown member of the putative species using the criterion that it must fall within, but not sister to, the (putative) species clade in a tree (P_ID(strict)_); (2) the probability that a putative species has the observed degree of distinctiveness due to random coalescent processes (P_RD_); and (3) the probability of reciprocal monophyly under a random coalescent model (Rosenberg’s PAB [46]). Because this method is applied to gene trees we chose 16S, the gene region for which we had the largest number of sequences (N = 55; Table 1) after incorporating recently published data [1], to compute an ultrametric (time) tree in Beast 1.8.3 (Yule speciation process; lognormal uncorrelated relaxed clock). We performed four independent runs for 10^7^ generations each, sampling every 1,000 generations. Results were analyzed in Tracer v1.6 [37] to assess convergence and effective sample sizes (ESS) for all parameters. After a 10% “burn-in”, trees were used to calculate posterior probabilities (PP) for each bipartition in a maximum clade credibility tree in TreeAnnotator v1.8.3 [38].

## Results

### Phylogeny and divergence times

**Fig 2:**
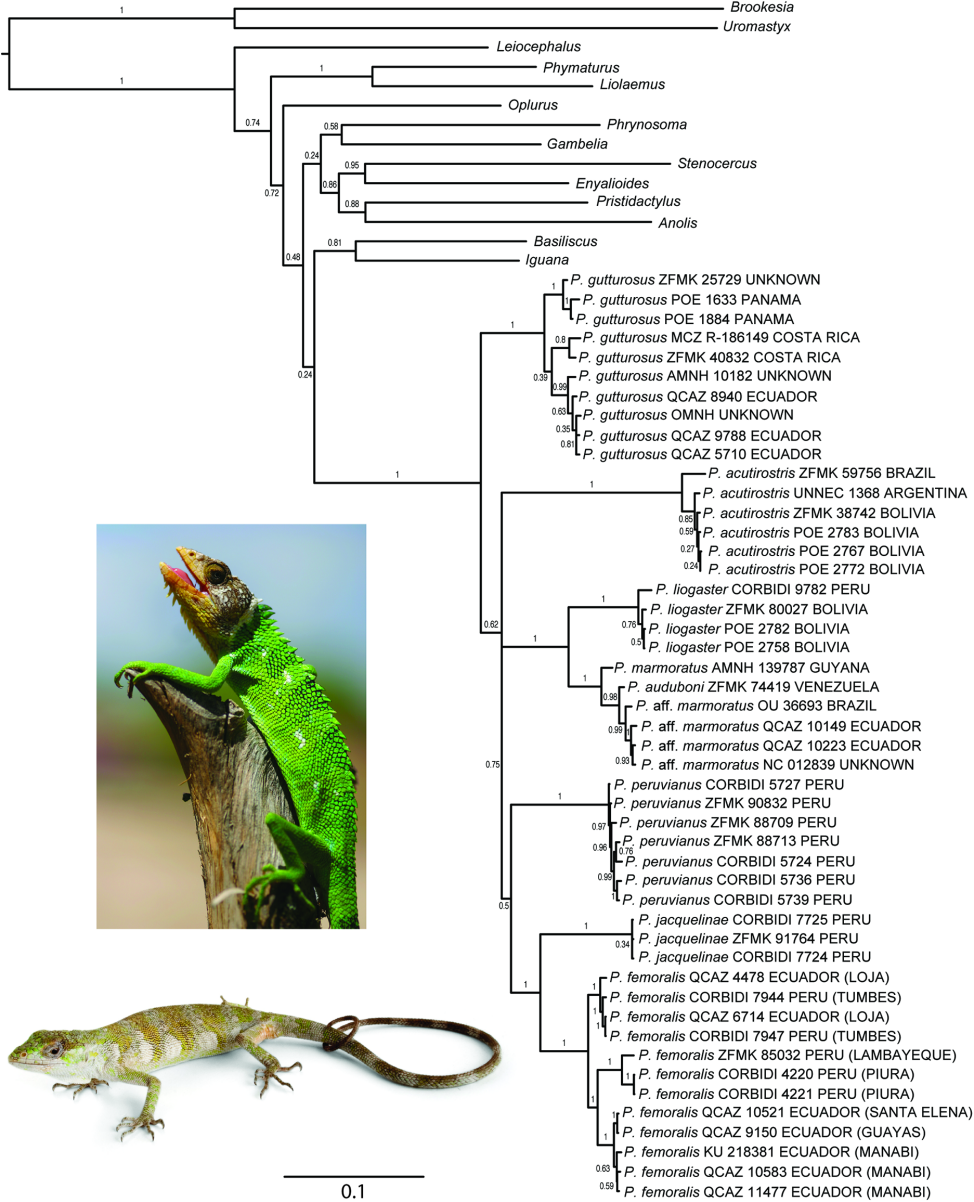
Phylogeny of iguanian lizards with emphasis on *Polychrus*. Maximum clade credibility tree obtained from a Bayesian analysis of 62 specimens, three mitochondrial genes (12S, 16S, ND2) and one nuclear gene (RAG1). Numbers above branches correspond to Bayesian posterior probability (PP) values. For specimens of *Polychrus*, voucher number and country of origin are indicated. GenBank accession numbers along with more detailed locality data are presented in Table 1 for all specimens included in this tree. Photographs: *P*. *peruvianus* (top; C. Koch), *P*. *femoralis* (bottom; F. Ayala-Varela).

**Fig 3:**
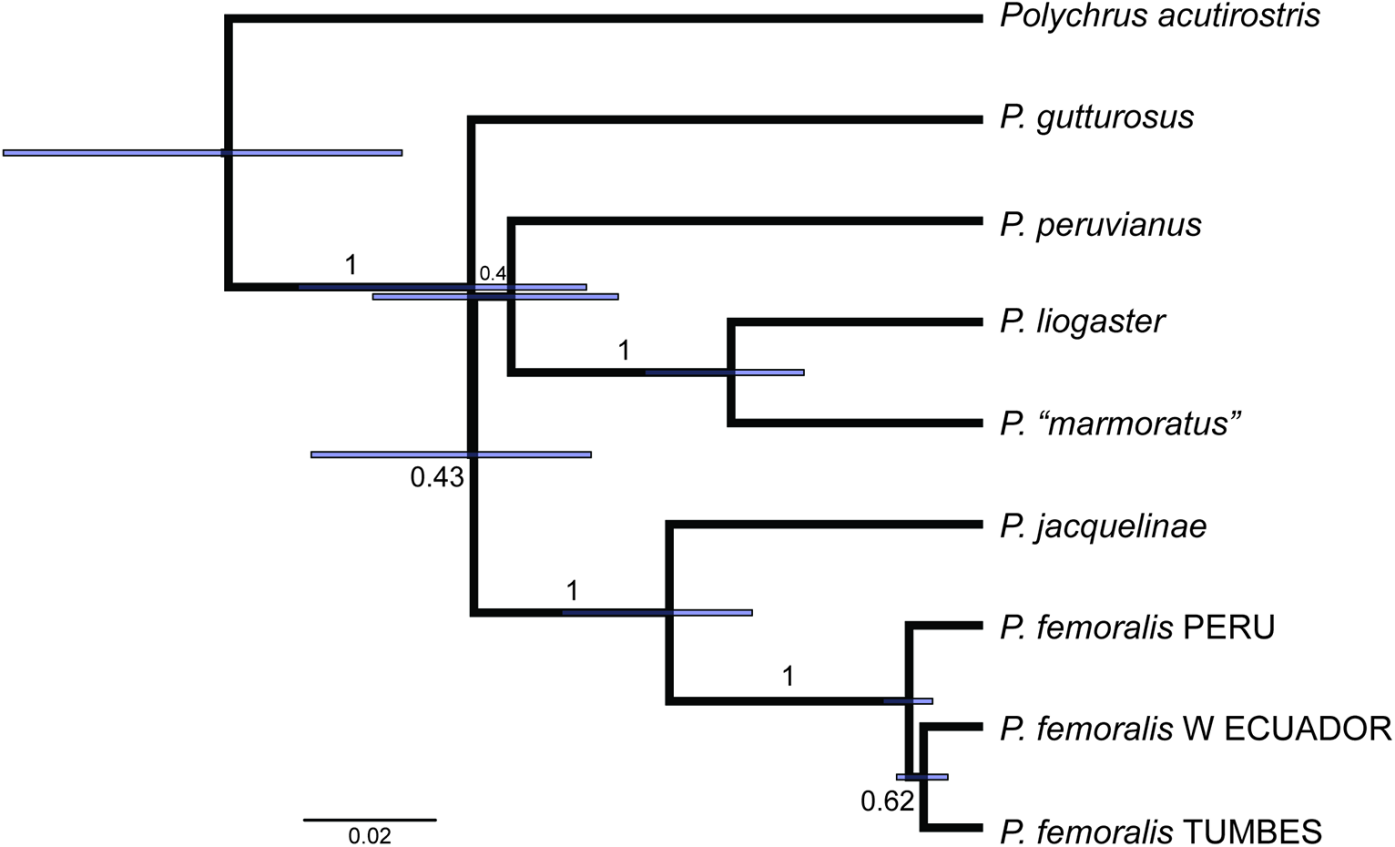
*Polychrus* species tree. Maximum clade credibility tree obtained from a Starbeast analysis of 31 specimens, three mitochondrial genes (12S, 16S, ND2) and one nuclear gene (RAG1). Numbers above branches correspond to Bayesian posterior probability (PP) values. Branch lengths units are expected substitutions per site.

Monophyly of *Polychrus* is strongly supported (PP = 1) by the concatenated gene tree (CGT), which includes representatives of most major iguanid lineages (Fig 2). This tree is similar in topology to the species tree (SPT; Fig 3) in that it strongly supports (PP = 1) a sister taxon relationship between *P*. *marmoratus* and *P*. *liogaster* and between *P*. *femoralis* Werner, 1910 and *P*. *jacquelinae*. Nonetheless, CGT and SPT have two major differences. First, according to the CGT, *P*. *gutturosus* is sister to all other species of *Polychrus*, which are clustered in a weakly supported (PP = 0.62) clade, where *P*. *acutirostris* Spix, 1825 is sister to a clade (PP = 0.75) composed of two subclades—(*P*. *marmoratus*, *P*. *liogaster*) with PP = 1, and ((*P*. *peruvianus*, (*P*. *femoralis*, *P*. *jacquelinae*)) with PP = 0.50. In contrast, the SPT has *P*. *acutirostris* as sister to a strongly supported clade (PP = 1) containing all other species of *Polychrus*. In this clade, *P*. *gutturosus* is sister to a clade (PP = 0.43) composed of two subclades—(*P*. *peruvianus*, (*P*. *marmoratus*, *P*. *liogaster*)) with PP = 0.40, and (*P*. *femoralis*, *P*. *jacquelinae*).

The SPT also shows that the split between the Pacific-western Andean species *Polychrus femoralis* and Amazonian *P*. *jacquelinae* occurred later than the split among Amazonian *P*. *peruvianus*, *P*. *marmoratus* and *P*. *liogaster*. In addition, the two splits corresponding to the three putative species currently recognized as *P*. *femoralis* (see below) are more recent (Fig 3). In contrast to the CGT, where the "*femoralis* putative species" from the Tumbes region (i.e., extreme northwestern Peru and southern Ecuador) is sister (PP = 1) to the other two putative species, in the SPT the "species" from northern Peru is sister to the clade (PP = 0.62) formed by the two "species" from the Tumbes region and western Ecuador.

Selected partitions and models of evolution for the CGT analysis are (i) 12S +16S (GTR + G); (ii) ND2, 1^st^ codon position (GTR + I + G); (iii) ND2, 2^nd^ codon position (GTR + I + G); (iv) ND2, 3^rd^ codon position (GTR + I + G); (v) RAG1, 2^nd^ and 3^rd^ codon positions (HKY + G); and (vi) RAG1, 1^st^ codon position (HKY + G). For the restricted dataset used in the SPT analysis the partition scheme is (i) 12S +16S (GTR + G); (ii) ND2, 1^st^ codon position (HKY + G); (iii) ND2, 2^nd^ codon position (HKY + G); (iv) ND2, 3^rd^ codon position (HKY + G); (v) RAG1, 2^nd^ and 3^rd^ codon positions (HKY); and (vi) RAG1, 1^st^ codon position (K80).

### Species diversity

**Fig 4:**
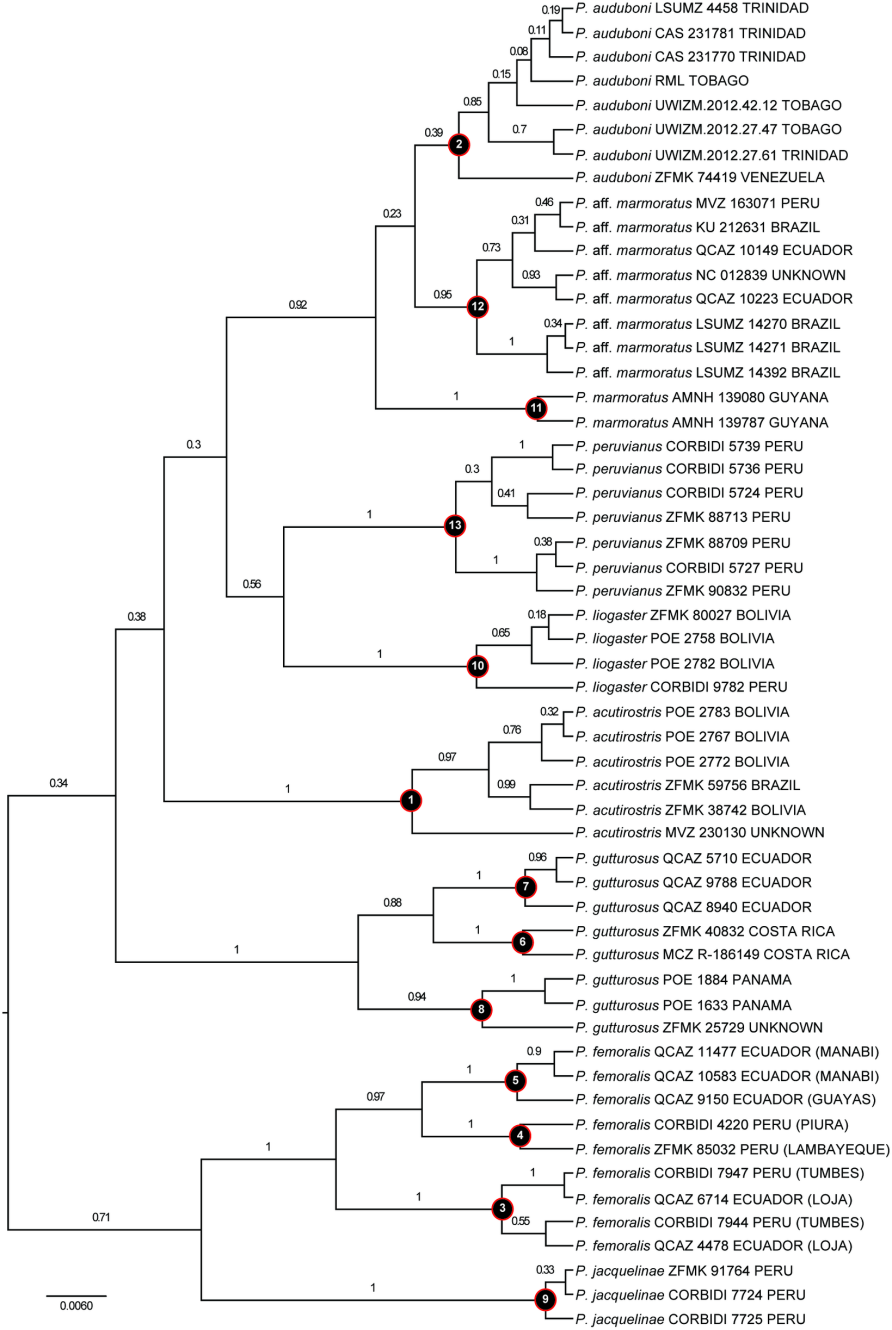
16S gene tree of *Polychrus*. Maximum clade credibility tree obtained from a Starbeast analysis of 55 specimens. Numbers above branches correspond to Bayesian posterior probability (PP) values. Taxon name, voucher number, and locality are indicated for each terminal. Node numbers correspond to both currently recognized and putative species as indicated in Table 4.

All currently recognized species of *Polychrus* were strongly supported (PP = 1) as monophyletic groups by the CGT (Fig 2). Based on relative branch lengths in the 16S ultrametric gene tree, geographic distribution, and a recent proposal of species delimitation within "*P*. *marmoratus*" [1], we identified the following lineages as putative separate species (Table 4, Fig 4): (1) *P*. *gutturosus* from Costa Rica, (2) *P*. *gutturosus* from Panama, (3) *P*. *gutturosus* from Ecuador, (4) *P*. *marmoratus* from Guyana, (5) *P*. *marmoratus* from the Amazon (Brazil, Ecuador and Peru), (6) *P*. *femoralis* from western Ecuador, (7) *P*. *femoralis* from Peru, and (8) *P*. *femoralis* from the Tumbes region in extreme northwestern Peru and southwestern Ecuador. Both "*P*. *marmoratus* from the Amazon" and "*P*. *femoralis* from the Tumbes region" had P_ID(strict)_ values (0.72) falling within the range of values (0.71–0.80) calculated for those species that were not "split" into putative species (i.e., *P*. *acutirostris*, *P*. *auduboni*, *P*. *jacquelinae*, *P*. *liogaster*, *P*. *peruvianus*). Other putative species had P_ID(strict)_ values below 0.61. Regarding Rosenberg’s P_AB_ statistic, "*P*. *marmoratus* from Guyana" (7.6E-4) and "*P*. *marmoratus* from the Amazon" (1.0E-5) had values falling within the range of observed values for unsplit species (3.6E-8–8.2E-4). Other putative species had Rosenberg’s P_AB_ values ranging between 0.01 and 0.05, except for "*P*. *femoralis* from the Tumbes region" (1.98E-03).

**Table 4:** Summary of results of the species delimitation analysis.

Taxon	N	D_intra_	D_inter_	D_intra_ / D_inter_	P_ID(strict)_	Rosenberg’s P_AB_
1: *P*. *acutirostris*	6	0.019	0.083	0.23	0.78 (0.65, 0.90)	3.6E-8
2: *P*. *auduboni*	8	0.015	0.032	0.47	0.71 (0.60, 0.81)	1.0E-5
3: *P*. *femoralis* Tumbes	4	0.011	0.048	0.23	0.72 (0.57, 0.86)	1.98E-03
4: *P*. *femoralis* Peru	2	0.011	0.031	0.35	0.41 (0.26, 0.56)	0.05
5: *P*. *femoralis* Ecuador	3	0.009	0.031	0.29	0.60 (0.42, 0.78)	0.05
6: *P*. *gutturosus* Costa Rica	2	0.010	0.028	0.36	0.41 (0.25, 0.56)	0.05
7: *P*. *gutturosus* Ecuador	3	0.008	0.028	0.27	0.61 (0.43, 0.79)	0.05
8: *P*. *gutturosus* Panama	3	0.014	0.044	0.33	0.57 (0.40, 0.75)	0.01
9: *P*. *jacquelinae*	3	0.004	0.076	0.06	0.75 (0.58, 0.93)	8.2E-4
10: *P*. *liogaster*	4	0.013	0.059	0.23	0.71 (0.57, 0.86)	6.0E-4
11: *P*. *marmoratus* Guyana	2	0.007	0.040	0.18	0.50 (0.35, 0.65)	7.6E-4
12: "*P*. *marmoratus"* Amazon	5	0.014	0.032	0.45	0.72 (0.61, 0.82)	1.0E-5
13: *P*. *peruvianus*	7	0.018	0.059	0.31	0.80 (0.69, 0.90)	6.0E-4

Taxon numbers are the same as those presented in the phylogenetic tree in Fig 4. The number of specimens per species (N); average pairwise tree distance among members of a putative species (D_intra_); average pairwise tree distance between members of one putative species and members of the closest second putative species (D_inter_); D_intra_/D_inter_ ratio; the mean (95% confidence interval) probability of correctly identifying an unknown member of the putative species using the criterion that it must fall within, but not sister to, the species clade in a tree P_ID(strict)_; the probability that a clade has the observed degree of distinctiveness due to random coalescent processes (P_RD_); and the probability of reciprocal monophyly under a random coalescent model (Rosenberg’s P_AB_) are presented.

## Discussion

### Phylogeny of *Polychrus* and divergence times

As expected by the relatively low number of characters and loci included in this study, the phylogenetic relationships among major lineages of iguanid lizards are poorly resolved (Fig 2); *Polychrus* is weakly supported (PP = 0.24) as sister to the clade (*Iguana*, *Basiliscus*). Whether *Polychrus* is sister to *Anolis* remains controversial (see Introduction). Nonetheless, in agreement with previous hypotheses [9, 10, 18], here we show that *Polychrus* is monophyletic based on phylogenetic analyses of the largest taxonomic and geographic sampling of *Polychrus* to date, including all species and samples from throughout the range of the clade. Despite our sampling effort, the relationships among species of *Polychrus* were only partially resolved. Frost et al. (2001) inferred *P*. *gutturosus* as sister to all other species of *Polychrus* recognized at the time (i.e., excluding *P*. *auduboni*, *P*. *jacquelinae*, *P*. *liogaster*, and *P*. *peruvianus*). Although our CGT weakly supports this relationship, our SPT strongly supports a different scenario where *P*. *acutirostris* is sister to all other species of *Polychrus* (Fig 3). In both CGT and SPT, the relationships among remaining species remain unclear except for the sister taxon relationship of both (*P*. *marmoratus*, *P*. *liogaster*) and (*P*. *jacquelinae*, *P*. *femoralis*). Moreover, no pair of sister species is strongly supported (i.e., all PP values ≤ 0.71) by the 16S gene tree (Fig 4).

The age of *Polychrus* has been estimated by several authors using different methods, as well as different taxon and character sampling strategies. These estimates are incongruent, ranging between ~32 and ~125 million years (Table 3). The limited taxon sampling of *Polychrus* (N = 1–4 species) and the lack of fossil calibrations within *Polychrus* in these studies evoke little confidence in any of these estimates and suggest that preference among them is arbitrary. In the absence of reliable calibration points, or reliable divergence time estimates, only arbitrary calibrations (e.g., ucld.mean fixed to 1.0) resulting in relative age estimates should be adopted. These estimates, however, still contain useful information on the relative timing of events (e.g., [47]). Based on the chronophylogenetic species tree analysis, here we conclude that the split between two species from west of the Andes occurred earlier than the split between two eastern Andean species, and that lineage divergence within *P*. *femoralis* is more recent. We refrain from drawing more time-related conclusions because they would be based on observations of poorly supported relationships (Figs 2 and 3).

### Biogeography of *Polychrus*

Given that our inferred phylogenies did not fully resolve the relationships among all species of *Polychrus* with high support, we refrained from carrying out phylogeny-based biogeographic analyses, such as ancestral area reconstruction. Nonetheless, our results provide a few insights into the biogeography of monkey lizards. First, the strongly supported position of *P*. *acutirostris* in the SPT (Fig 3) suggests that *Polychrus* has its origins in South America rather than Central America, because this species is presently widespread along the South American diagonal belt of open formations that goes from Argentina and Bolivia to northeastern Brazil, encompassing the Chaco, Cerrado, and Caatinga biomes [48]. Second, our hypotheses (Figs 2 and 3) do not support a basal split between species presently occurring west (*P*. *femoralis*, *P*. *gutturosus*) and east (all other species) of the Andes, suggesting that the orogeny of the Andes did not play a major role in the early evolution of *Polychrus*. Even though this is in agreement with most age estimates of *Polychrus* (Table 3), we believe that this biogeographic scenario should be tested more rigorously.

### Diversity of *Polychrus*

Monophyly and diagnosability according to DNA sequence data are commonly used lines of evidence in species delimitation. As species properties, however, they are neither infallible nor essential (i.e., their absence does not constitute evidence contradicting a hypothesis of lineage separation) [49]. In this paper we explored species limits within currently recognized species of *Polychrus* by calculating monophyly and diagnosability statistics on a 16S gene tree (Table 4).

A growing body of evidence suggests that the diversity of vertebrates from tropical South America is underestimated as widely distributed species usually represent species complexes, in which cryptic or poorly studied species await discovery [50–53]. Among species of *Polychrus*, *P*. *acutirostris* and *P*. *marmoratus* have large geographical ranges (Fig 1), which makes them suitable for species delimitation analyses. Cryptic diversity within *P*. *marmoratus* was recently reported by Murphy et al. (2017) on the basis of morphology and a phylogeny of two mitochondrial genes. They recognized populations from Trinidad, Tobago and northern Venezuela as a separate species, *P*. *auduboni*. Following Hoogmoed [54], Murphy et al. (2017) also restricted the name *P*. *marmoratus* to the populations in Guyana and Suriname (and possibly French Guyana and northern Brazil), and suggested that two additional species might occur in southeastern Brazil (see also [5]). However, the phylogenetic position of populations from the Amazon region (Brazil and Peru) were not clearly resolved [1]. Here we present phylogenies with better resolution (Figs 2–4), which along with the results of the species delimitation analyses (Table 4), support recognition of *P*. *auduboni* and restriction of *P*. *marmoratus* to Guyana and Suriname. These taxonomic changes leave populations of "*P*. *marmoratus*" from the Amazon region in need of a different specific name. Populations of "*P*. *marmoratus*" from southeastern Brazil might also represent different species, for which the names *P*. *virescens* Schniz, 1822 and *P*. *neovidanus* Wagler 1833 are available [1]. However, here we refrain from proposing additional taxonomic changes because we believe that both denser molecular and geographical sampling, as well as detailed morphological analyses are necessary to elucidate more objectively the taxonomic status of other populations traditionally assigned to *P*. *marmoratus*, as well as *P*. *acutirostris*.

Among species with more restricted distribution ranges, neither the P_ID(strict)_ or Rosenberg’s P_AB_ statistics supported recognition of any of the three subclades within *P*. *gutturosus* (Panama, Costa Rica, and Ecuador; Fig 4) as separate species. In contrast, the same statistics suggest that populations of *P*. *femoralis* from the Tumbes region might belong to a cryptic undescribed species. If additional lines of evidence support this hypothesis (C. Koch, O. Torres-Carvajal and P.J. Venegas, unpubl. data), the name *P*. *femoralis* should be restricted to populations from western Ecuador based on type locality (Guayaquil, Ecuador). In this case, the disjunct set of populations from the Pacific slopes of the Andes in northern Peru (Piura and Lambayeque departments, Fig 4) could be either conspecific with *P*. *femoralis*, or represent a distinct undescribed species. We have eschewed describing new taxa in this paper, as our aim was to provide a general framework for future studies. Additional lines of evidence will lead to a better informed species delimitation process for Neotropical monkey lizards.
